# Assessment of oligometastasis status of prostate cancer following combined robot-assisted radical prostatectomy and androgen deprivation versus androgen deprivation therapy alone using PSA percentage decline rate

**DOI:** 10.3389/fendo.2023.1123934

**Published:** 2023-02-09

**Authors:** Xuwen Li, Haibo Xi, Xiaofeng Cheng, Yue Yu, Cheng Zhang, Gongxian Wang, Xiaochen Zhou

**Affiliations:** Department of Urology, The First Affiliated Hospital of Nanchang University, Nanchang, China

**Keywords:** oligometastatic prostate cancer, androgen deprivation therapy, tobot-assisted radical prostatectomy, TPSA, PSA percentage decline rate

## Abstract

**Objective:**

To compare the tumor control in prostate cancer patients with oligo-metastasis following combined robot-assisted radical prostatectomy and androgen deprivation versus androgen deprivation therapy alone based on total prostate-specific antigen (tPSA) assessment.

**Methods:**

Medical data of a total of 18 prostate cancer patients with oligometastasis administered in The First Affiliated Hospital of Nanchang University from March 2017 to March 2018 were prospectively collected. 10 patients received a combined therapy of robot-assisted radical prostatectomy and pharmaceutical androgen deprivation (RARP+ADT group), while 8 patients received pharmaceutical androgen deprivation therapy alone (ADT group). Then demographic characteristics, prostate volume, tumor characteristics and tPSA data were analysised and compared. Statistical analysis was performed using t-test for continuous variables and Pearson chi-square test or Fisher’s exact test for categorical variables.

**Results:**

No significant difference was found in patients’ age (*p* = 0.075), prostate volume (*p* = 0.134) and number of bone metastasis (*p* = 0.342). Pre-treatment Gleason score was significantly lower in RA group (*p* = 0.003). Patients in RARP+ADT group had significantly lower pre-treatment tPSA (*p* = 0.014), while no statistical difference was noted in reexamined tPSA (*p* = 0.140) on follow-up. No statistical difference was noted in tPSA decline rates (declined tPSA value per day) in RARP+ADT and ADT group (8.1 ± 4.7 verse 7.5 ± 8.0 ng/ml/d, *p* = 0.853). However, tPSA percentage decline rate (declined tPSA percentage per day) was significantly higher in RARP+ADT group (11.6 ± 1.5%/d verses 2.9 ± 2.2%/d, *p*< 0.001). Immediate urinary continence was achieved in 9 patients (90%) upon removal of urethral catheter on post-operative day 7 in RARP+ADT group.

**Conclusion:**

ADT alone and in combination with RARP both provide effective tumor control in patients suffering from prostate cancer with oligometastasis. ADT combined with RARP exhibited significant advantage in PSA percentage decline rate without compromising patients’ urinary continence. Long-term tumor control requires further follow-up.

## 1 Introduction

Prostate Cancer (PCa) is one of the most common malignant tumor of the male reproductive system, with one of the highest morbidity rate in European and American countries (1), and the second highest mortality rate, after lung cancer (2). The incidence of PCa has increased significantly in recent years in our country. Patients with localized PCa have an excellent prognosis after radical prostatectomy, whereas patients with metastatic PCa have an average survival time of only 42.1 months (3). Unfortunately, the proportion of patients with late partial or distant metastasis in our country is as high as 60 ~ 70% (4). As a result, improving tumor control in patients with metastatic PCa is a critical issue that must be addressed urgently in clinic.

In the mid-1990s, Hellman and Weichselbaum jointly proposed the concept of “oligometastases” in tumors, and described the oligometastases state as a period of mild tumor bioaggressivity, a transitional stage between localized disease and widespread metastasis. The number of metastatic tumors is limited and the metastatic organs are limited and qualitative, and have not been disseminated throughout the body (5). While androgen deprivation therapy (ADT) is considered the first-line treatment regimen in EAU guidelines for patients diagnosed with metastatic PCa for the first time, a number of studies have shown that patients with metastatic PCa who received both local treatment (including radical prostatectomy and radical radiotherapy) and ADT had significant advantages in overall survival, tumor-specific survival, progression-free survival, and progression to castration-resistant PCa (6–9). Nonetheless, in patients with metastatic PCa, local therapy combined with ADT is not part of the standard treatment protocol.

Therefore, we conducted this study to compare the short-term tumor control effects of robot-assisted radical prostatectomy combined with androgen deprivation therapy (RARP+ADT) versus ADT alone. We adopted total prostate-specific antigen (tPSA) as a reference indicator. We hope that this study will provide more data and evidence for the clinical value of local therapy in the control of oligometastatic PCa tumors.

## 2 Materials and methods

### 2.1 Data source and ethics statement

After obtaining the approval from the institutional review board and ethics committee of the First Affiliated Hospital of Nanchang University, we prospectively collected demographic and clinical data of patients with oligo-metastatic PCa admitted between March 2017 and March 2018.

### 2.2 Patient selection

Patients with elevated tPSA and suspected PCa were identified as potential candidates. All potential candidates underwent systemic imaging and prostate biopsy prior to treatment. Only when a prostate biopsy confirmed a prostate cancer and imaging examination suggested bone metastasis, these potential candidates were listed as official candidates. Patients over the age of 85 and those with radiographic evidence of visceral metastasis were excluded. Prior to treatment, candidates were informed of all treatment options including surgery and/or ADT. All candidates were informed that ADT was compulsory and strongly recommended. They were also told that concomitant RARP might yield better overall tumor control result than receiving ADT alone. Patients that agreed to RARP were then assigned to RARP+ADT group, while the otherwise were assigned to ADT group. Informed consent forms were obtained from all candidates prior to treatment. Eventually, a total of 18 patients with oligo-metastatic PCa were enrolled. Ten patients were treated with robot-assisted radical prostatectomy and androgen deprivation while 8 patients were managed by androgen deprivation therapy alone.

### 2.3 Technical considerations

In the combination treatment group, patients were scheduled to receive robot-assisted radical prostatectomy and androgen deprivation therapy (RARP+ADT). All patients received oral Bicalutamide (50 mg qd, AstraZeneca) from the day before surgery and subcutaneous Goserelin Acetate (3.6 mg qm, AstraZeneca) from the fifth day after surgery. All operations were performed by the same experienced medical team.

In the ADT group, Bicalutamide (50 mg qd, AstraZeneca) was taken orally from the first day of treatment, and Goserelin Acetate (3.6 mg qm, AstraZeneca) was injected subcutaneously 6 days later.

### 2.4 Objectives and data collection

#### 2.4.1 Objectives

Primary objective of the study was to assess the PSA percentage decline rate between patients that received RARP+ADT and those treated by ADT alone, which was calculated as $\frac{\text{pre}-\text{tPSA}-\text{pos}-\text{tPSA}}{\text{pre}-\text{tPSA}}\times 100\%÷\text{tPSA}\;\text{review}\;\text{interval}$ Pre-tPSA was the baseline total PSA obtained prior to treatment; pos-tPSA was the total PSA obtained after a certain time following treatment; tPSA review interval is the time gap between the time of initial treatment and the time of first tPSA following treatment. The secondary objective was to assess the tPSA decline rate between the two group of patients, which was calculated as $\frac{\text{pre}-\text{tPSA}-\text{pos}-\text{tPSA}}{\text{tPSA}\;\text{review}\;\text{interval}}$ .

#### 2.4.2 Data collection

Pre-treatment assessment included age, body mass index (BMI), prostate volume, tumor characteristics (Gleason score, bone metastasis and distant lymph node metastasis), urinary control (assessed by ICI-Q-SF) and pre-tPSA. The post-treatment assessment was the pos-tPSA data and interval at the first review. Both intraoperative and postoperative conditions (operation time, blood loss, complications, catheter removal time, urinary control) were also recorded in the RARP+ADT group.

### 2.5 Statistical analysis

Means and standard deviations were determined for the normally distributed continuous variables, while those with nonnormal distribution were presented as median and interquartile range. Categorical variables were presented as frequencies and their proportions. The Student’s t-test was performed for the normally distributed continuous variables. All categorical variables were compared with the Chisquare test. SPSS 26.0 (IBM Corp, Armonk, NY) was utilized for all statistical analysis with a two-sided p value < 0.05 denoting statistical significance.

## 3 Results

A total of 18 candidates were included in the study between March 2017 and March 2018. These candidates were divided into two groups, the RARP+ADT group (n = 10) and the ADT group (n = 8). Demographic characteristics, prostate volume, and tumor characteristics are shown in **Table 1**. The age was 70.9 ± 6.9 yrs (mean ± SD) ranged from 61.0 to 82.0 yrs in ADT group versus 64.6 ± 7.0 yrs ranged from 56.0 to 77.0 yrs in RARP+ADT group, respectively (*p* = 0.075). The weight was 68.4 ± 6.2 kg ranged from 59.0 to 80.0 kg in ADT group versus 70.4 ± 7.4 kg ranged from 59.0 to 80.0 kg in RARP+ADT group, respectively (*p* = 0.545). The BMI was 25.7 ± 3.0 kg/m^2^ ranged from 22.5 to 31.3 kg/m^2^ in ADT group, while the RARP+ADT group was 25.1 ± 7.5 kg/m^2^ ranged from 21.7 to 29.4 kg/m^2^, respectively (*p* = 0.715). Prostate volume was 44.8 ± 21.9 cm^3^ ranged from 20.5 to 91.1 cm^3^ in ADT group versus 57.3 ± 11.4 cm^3^ ranged from 41.4 to 72.4 cm^3^ in RARP+ADT group, respectively (*p* = 0.134). Gleason score was 8.6 ± 0.9 ranged from 8.0 to 10.0 in ADT group, while the RARP+ADT group was 7.3 ± 0.7 ranged from 6.0 to 8.0, respectively (*p* = 0.003). The bone metastasis was 3.0 ± 1.1 ranged from 2.0 to 5.0 in ADT group, while 2.5 ± 1.1 ranged from 1.0 to 5.0 in RARP+ADT group, respectively (*p* = 0.342). All candidates had normal urine control before treatment.

**Table 1:** Demographic characteristics, prostate volume and tumor characteristics.

	ADT (n = 8)	RARP+ADT (n = 10)	*p*
Age (yr), mean (SD), range	70.9 (6.9), 61.0 - 82.0	64.6 (7.0), 56.0 - 77.0	0.075
Weight (kg), mean (SD), range	68.4 (6.2), 59.0 - 80.0	70.4 (7.4), 59.0 - 80.0	0.545
BMI (kg/m^2^), mean (SD), range	25.7 (3.0), 22.5 - 31.3	25.1 (7.5), 21.7 - 29.4	0.715
Prostate volume (cm^3^), mean (SD), range	44.8 (21.9), 20.5 - 91.1	57.3 (11.4), 41.4 - 72.4	0.134
Gleason score, mean (SD), range	8.6 (0.9), 8.0 - 10.0	7.3 (0.7), 6.0 - 8.0	0.003
Bone metastasis, mean (SD), range	3.0 (1.1), 2.0 - 5.0	2.5 (1.1), 1.0 - 5.0	0.342
Distant lymph node metastasis, mean (SD), range	0	0	–
Urinary control, n (%)	8 (100.0%)	10 (100.0%)	–

**Table 2** records the intraoperative and postoperative conditions of patients in RARP+ADT group. All operations were successfully completed. The operation time was 113.5 ± 14.7 min ranged from 90.0 to 140.0. The blood loss was 88.0 ± 53.5 ml ranged from 40.0 to 200.0 ml. Two patients developed fever and managed with antibotics (Clavien-Dindo Grade II). All patients had their catheters removed 7 days after surgery. One patient had urinary incontinence after catheter removal.

**Table 2:** Perioperative data of RARP+ADT group.

	RARP+ADT (n = 10)
Operation time (min), mean (SD), range	113.5 (14.7), 90.0 - 140.0
Blood loss (ml), mean (SD), range	88.0 (53.5), 40.0 - 200.0
Complications	
Fever, n (%)	2 (20.0%)
Leaking urine, n (%)	1 (10.0%)
Urinary control, n (%)	9 (90.0%)

All patients were monitored for tPSA before and after treatment and followed up for at least 4.5 years. The pre-tPSA was 235.3 ± 144.0 ng/ml ranged from 56.7 to 420.0 ng/ml in ADT group, which was significantly higher than RARP+ADT group (70.6 ± 42.9 ng/ml ranged from 18.3 to 134.6 ng/ml, *p* = 0.014). The pos-tPSA was 35.9 ± 42.4 ng/ml ranged from 0.2 to 129.6 ng/ml in ADT group versus 14.1 ± 12.4 ng/ml ranged from 1.3 to 43.8 ng/ml in RARP+ADT group, respectively (*p* = 0.140). The tPSA review interval was 44.0 ± 27.4 days ranged from 13.0 to 90.0 days in ADT group, while all patients in the RARP+ADT group received tPSA re-examination on the 7th day after surgery. The tPSA decline rate were comparable between two groups (7.5 ± 8.0 ng/ml/d vs. 8.1 ± 4.7 ng/ml/d, *p* = 0.853), while the PSA percentage decline rate was significantly different (2.9 ± 2.2%/d vs. 11.6 ± 1.5%/d, *p*< 0.001, **Table 3**).

**Table 3:** tPSA decline rate and tPSA percentage decline rate.

	ADT (n = 8)	RARP+ADT (n = 10)	*p*
pre-tPSA^a^ (ng/ml), mean (SD), range	235.3 (144.0), 56.7 - 420.0	70.6 (42.9), 18.3 - 134.6	0.014
pos-tPSA^b^ (ng/ml), mean (SD), range	35.9 (42.4), 0.2 - 129.6	14.1 (12.4), 1.3 - 43.8	0.140
tPSA review interval^c^ (d), mean (SD), range	44.0 (27.4), 13.0 - 90.0	7.0	0.007
tPSA decline rate^d^ (ng/ml/d), mean (SD), range	7.5 (8.0), 1.0 - 24.6	8.1 (4.7), 2.4 - 15.0	0.853
PSA percentage decline rate^e^ (%/d), mean (SD), range	2.9 (2.2), 1.1 - 6.7	11.6 (1.5), 8.3 - 13.3	< 0.001

a , baseline total PSA obtained prior to treatment.

b , total PSA obtained after a certain time (tPSA review interval) following treatment.

c , tPSA review interval: the time gap between the time of initial treatment and the time of first tPSA following treatment. tPSA was re-examined 7 days after surgery in all patients in RARP+ADT group.

d , tPSA decline rate was calculated as $\frac{\text{pre}-\text{tPSA }-\text{ pos}-\text{tPSA}}{\text{tPSA review interval}}$ .

e , PSA percentage decline rate $\left(\text{PSAPDR}\right)=\frac{\text{pre}-\text{tPSA }-\text{ pos}-\text{tPSA}}{\text{pre}-\text{tPSA}}\times 100\%÷\text{tPSA review interval}$ .

## 4 Discussion

Genomic studies on oligometastatic PCa have indicated its unique biological characteristics, suggesting that it may be a special subtype of PCa (10–13). Although the concept of oligometastases was proposed as early as in the 1990s (5), there is no standard definition of the scope and number of lesions of oligometastases at present. Through literature search, we found that 6 papers defined the number and range of metastases of oligometastases: two of them took ≤ 5 metastases as the standard (14, 15), One paper was based on ≤ 4 metastases (16), and in three papers, the standard was ≤ 3 metastases (17–19). Two studies limited the metastases to bone metastases and distant lymphatic metastases (17, 18).

Accumulating evidence suggested that patients with oligometastatic PCa might benefit from the surgical removal of the cancerous prostate gland. There have been several clinical studies on the surgical treatment of metastatic PCa. SWOG (Southwest Oncology Group) study No. 8894 reviewed 1286 patients with PCa with bone metastasis, and the results suggested that based on ADT, the overall survival rate of patients undergoing surgery was 1.55 times that of those without surgery (20). Heidenreich reported 61 cases of oligometastatic PCa with an average follow-up of > 40 months. The results showed that the overall survival rates of patients with surgery combined with ADT and ADT alone were 91.3% and 78.9%, respectively, and the tumor specific survival rates were 95.6% and 84.2%, respectively, with significant differences (8). Culp reviewed 8185 cases of metastatic PCa from the U.S. National Cancer Institute’s SEER (Surveillance Epidemiology and End Results) database, followed for an average of 16 months. The overall survival rates of patients treated with ADT combined with surgery, ADT combined with radiotherapy and ADT alone were 64.7%, 62.6% and 22.5%, respectively, and the tumor-specific survival rates were 75.8%, 61.3% and 48.7%, respectively, suggesting that surgery and radiotherapy brought significant survival benefits to patients compared with no local therapy (6). Similar results were also obtained in the retrospective comparative analysis of 7858 patients with metastatic PCa by Antwi (7). Locally advanced and inoperable patients can be treated with radiotherapy for local tumor control. However, Gratzke et al. reviewed 1538 cases of metastatic PCa included in the Missouri Cancer Registry (MCR) database. The results suggest that surgery is significantly better than radiotherapy, ADT alone or other treatments in terms of overall survival (21). Similar results were also obtained in a retrospective analysis of 916 cases of metastatic PCa conducted by Taipei Medical University and New Jersey Cancer Institute (22). Only one prospective randomized controlled study evaluating the efficacy of surgical treatment for metastatic PCa has been reported, and the results showed that ADT combined with surgery has significant advantages over ADT alone in progression-free survival, overall survival, tumor-specific survival, and progression to castration-resistant PCa (8). There are two popular theories about the relationship between primary tumor and metastasis, namely “seed and soil” theory and “self-planting” theory. The former refers to the factors secreted by the tumor primary site, which can promote the microenvironment (soil) suitable for the growth of circulating tumor cells (seeds) in other parts of the body (23). The latter refers to the fact that tumor cells from distant metastases can be replanted in the primary site, leading to a vicious cycle between the primary site and the metastases (24, 25). Therefore, removal of the primary site may interrupt the complementary relationship between the primary site and the metastatic site, which is beneficial for tumor control.

Serum PSA is the preferred screening indicator for PCa and the main tumor marker for prognosis assessment. In addition to the PSA value itself, PSA dynamics is also of great value for diagnosis and prognosis assessment. Currently, PSA dynamics consists of three reference criteria, namely PSA velocity (PSAV), PSA doubling time (PSADT), and PSA flare phenomenon (PSAFP). The concept of PSAV is based on the linear relationship between PSA growth and time. The calculation formula of PSAV in AJCC Guide 2014 edition is PSAV = [(PSA2 - PSA1) + (PSA3 - PSA2)] ÷ 2, where PSA1, PSA2 and PSA3 are the results of three PSA tests within two years. Studies have shown that PSAV > 2 ng/ml/year indicates that surgical treatment may be necessary for patients (26). The concept of PSADT is based on the power function relationship between PSA growth and time, which is a descriptive indicator of the rate of PSA re-increase in PCa patients after treatment. It also contains two aspects of basic PSA level and PSAV information. The calculation formula is PSADT = (t2 - t1) lg2 ÷ (lgPSA2 - lgPSA1). A large retrospective study stratified PSADT by< 3 months, 3 to 8 months, 9 to 14 months, and > 15 months suggested a significant correlation between PSADT and tumor-specific and overall survival, and patients with PSADT< 9 months had a poor prognosis (27). The AJCC guidelines states that PSADT is useful for assessing local recurrence and distant metastasis. PSAFP refers to the abnormal rise of PSA in patients with advanced PCa after the start of second-line therapy (such as paclitaxel-based chemotherapy regimen and abiraterone endocrine therapy), which soon drops below the baseline level (28). There is increasing evidence that the occurrence of this phenomenon may indicate that patients are responding to treatment (29).

**Figure 1:**
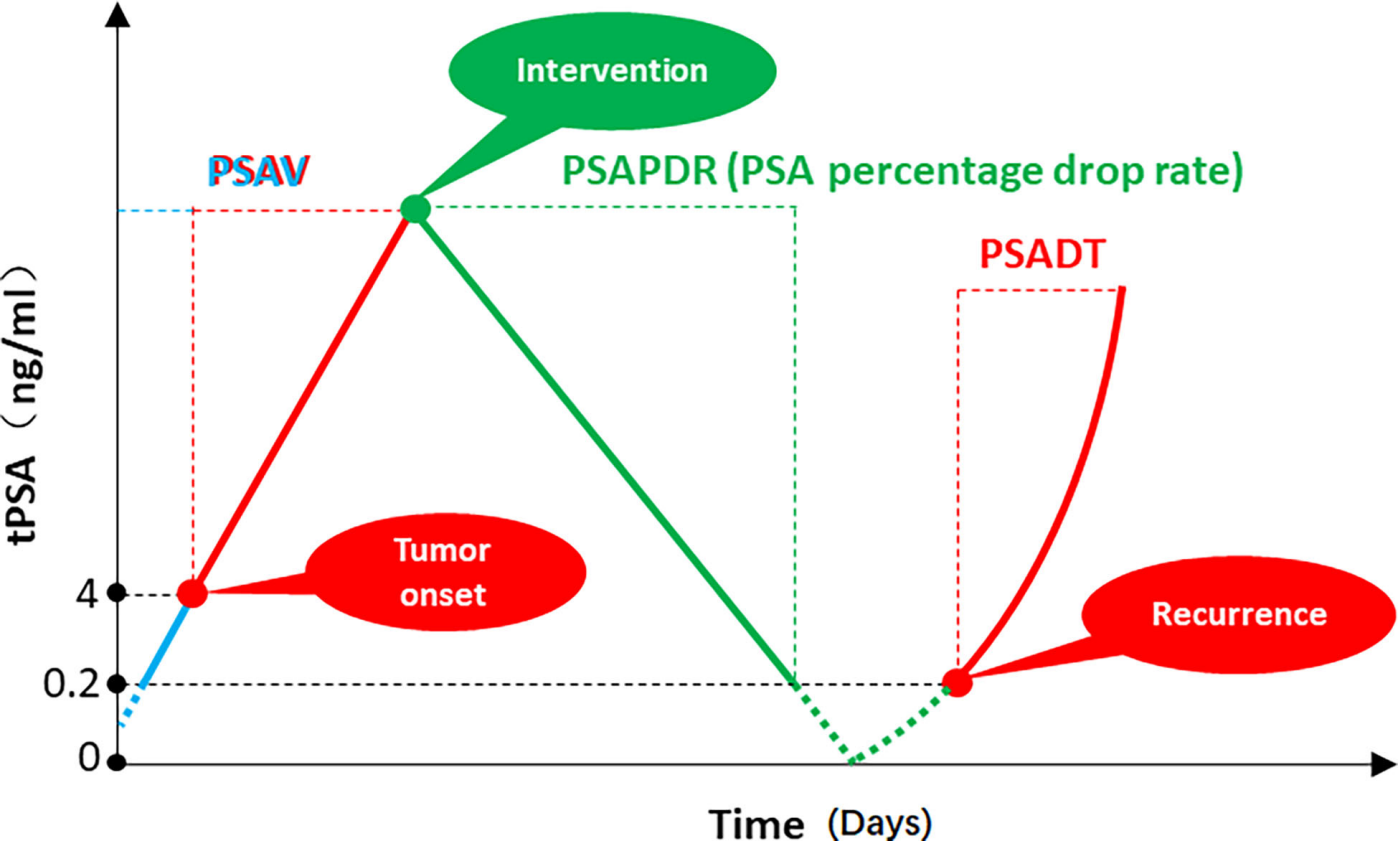
PSA velocity (PSAV), PSA doubling time (PSADT), and PSA percentage decline rate (PSAPDR). The concept of PSAV is based on the linear relationship between PSA growth and time. The calculation formula is [(PSA2 - PSA1) + (PSA3 - PSA2)] ÷ 2. The concept of PSADT is based on the power function relationship between PSA growth and time, which is a descriptive indicator of the rate of PSA re-increase in PCa patients after treatment. (t2 - t1) lg2 ÷ (lgPSA2 - lgPSA1). PSA percentage decline rate (PSAPDR) was defined as the percentage of PSA decline in unit time (days as unit time), which calculated as [(initial PSA- reexamination PSA)] ÷ initial PSA × 100% ÷ days between two PSA examinations.

With the occurrence and development of prostate cancer, the PSA of patients usually keeps rising in a certain period of time. Following ADT and/or local therapy, PSA typically continues to decrease over time up to baseline levels that are correlated with the patient’s tumor load and treatment, with some individualized variation. When treatment fails and the tumor continues to progress, PSA may show a trend of continuous increase (**Figure 1**). As mentioned above, PSAV, a concept based on the linear relationship between PSA growth and time, can be used to reflect the stage of tumor occurrence and development. From a mathematical point of view, this value normalized the time of two PSA reviews and the absolute value of PSA used to calculate PSAV. That is, on the assumption of a linear relationship between PSA growth and time, PSAV is still comparable among different patients, different initial PSA and different review time. The upper limit of reference value can be obtained through correlation studies (for example, PSAV > 2 ng/ml/year indicates that surgical treatment may be necessary for patients (26)). PSADT, a concept based on the power function relationship between PSA growth and time, is used to reflect the rise rate of PSA during the period of tumor progression after treatment. From a mathematical point of view, this value is also standardized for PSA interval time and absolute value of PSA used to calculate PSADT. PSADT values calculated by any two points on the left red line were the same (**Figure 1**). That is, PSADT was also comparable among different patients, different initial PSA and different review time based on the assumption that the change trend of PSA was a power function with time. The upper limit of reference value can also be obtained through correlation studies (for example, patients with PSADT< 9 months have poor prognosis (27)). However, there is currently a lack of reference indicators similar to PSAV and PSADT to describe the rate of PSA decline after treatment.

Considering that the time of PSA review after treatment may be different for different patients, and the initial PSA may be different, we proposed the PSA percentage decline rate (PSAPDR) based on the concept of a linear relationship between PSA decline and time. That is, the percentage of PSA decline in unit time (days as unit time), calculated by PSAPDR = [(initial PSA- reexamination PSA)] ÷ initial PSA × 100% ÷ days between two PSA examinations. PSAPDR normalized the initial PSA with “percentage” and the review time with “rate of decline”. In other words, although the absolute value of PSA (green dot) and the review time might be different among patients, the PSAPDR values calculated at any two points on the green solid line are the same (**Figure 1**). That is, similar to PSAV and PSADT, PSAPDR also has comparability among different patients who has different initial PSA and different review time. It is possible to obtain a reference value through subsequent correlation studies to evaluate the effect of a treatment on tumor control, and even to predict the risk of tumor recurrence and timely intervention.

In this study, PSAPDR in the RARP+ADT group and the ADT group were 11.6 ± 1.5%/d and 2.9 ± 2.2%/d, respectively, with significant statistical difference (*p<* 0.001), suggesting that the effect of surgery combined with ADT on PSA reduction was significantly higher than that of ADT alone. There was no significant difference in the tPSA decline rate between the RARP+ADT group and the ADT group, namely the absolute value of daily tPSA decline (8.1 ± 4.7 and 7.5 ± 8.0 ng/ml/d, respectively) (p = 0.853). This may be related to the significantly higher initial PSA in the ADT group than in the RARP+ADT group (235.3 ± 144.0 vs. 70.6 ± 42.9 ng/ml, p = 0.014).

In conclusion, robot-assisted radical prostatectomy combined with ADT and ADT alone are two effective treatments for oligometastatic PCa. Under the premise of strict control of surgical safety, taking PSAPDR (PSA percentage decline rate) as the reference index, surgery combined with ADT seems to have a better effect on the reduction of tPSA in patients than ADT alone. The main limitations of this study are the limited number of cases in the two groups involved and the short follow-up time. The clinical significance of PSAPDR in terms of patients’ survival (overall survival, progression-free survival and etc) requires further investigation.

## 5 Conclusions

In conclusion, robot-assisted radical prostatectomy combined with ADT and ADT alone are two effective treatments for oligometastatic PCa. Under the premise of strict control of surgical safety, taking PSAPDR (PSA percentage decline rate) as the reference index, surgery combined with ADT seems to have a better effect on the reduction of tPSA in patients than ADT alone. The main limitations of this study are the limited number of cases in the two groups involved and the short follow-up time. The clinical significance of PSAPDR in terms of patients’ survival (overall survival, progression-free survival and etc) requires further investigation.

## Data availability statement

The original contributions presented in the study are included in the article/**Supplementary Material**. Further inquiries can be directed to the corresponding authors.

## Ethics statement

The studies involving human participants were reviewed and approved by the Ethical Committee of The First Affiliated Hospital of Nanchang University. The patients/participants provided their written informed consent to participate in this study.

## Author contributions

Conception and design: GW and XZ. Surgeons: GW and XZ. Acquisition of data: XL. Preparation of tools: YY and CZ. Analysis and interpretation of data: XC. Drafting of the manuscript and statistical analysis: XL. Critical revision: GW and HX. Obtaining funding: XZ and HX. All authors contributed to the article and approved the submitted version.
